# Supplementary material for: An AC-Rich Bean Element Serves as an Ethylene-Responsive Element in Arabidopsis
Source: Plants (Basel). 2020 Aug 14;9(8):1033. doi: 10.3390/plants9081033 (PMC7465537; doi:10.3390/plants9081033)
Supplement: Supplementary file 1 [file plants-09-01033-s001.pdf]

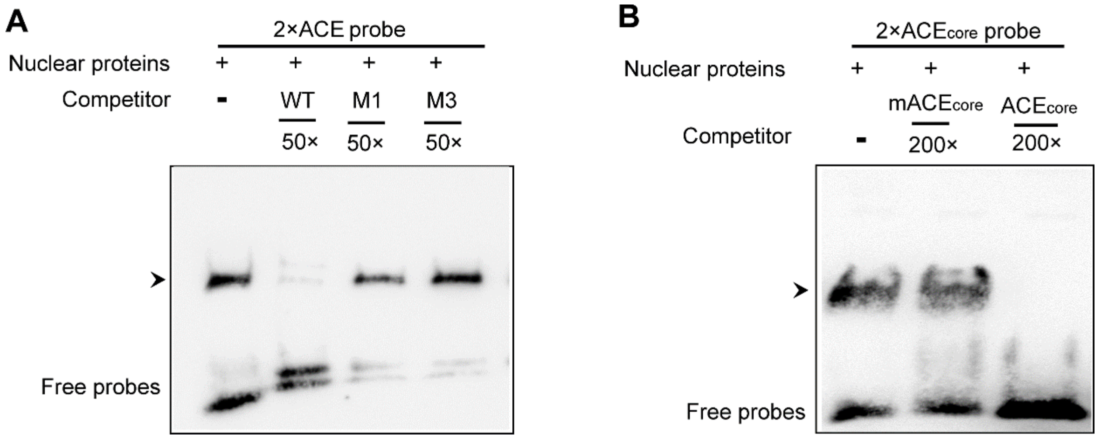


**Figure S1. The repeat EMSAs confirming the results in Figure 3.**

**(A)** ACE (2×) was used as the probe in the competitive EMSA with the nuclear proteins, and the wild-type ACE (WT) and two ACE variants (M1 and M3) were used as competitors (50-fold molar). **(B)** The $ACE_{core}$/nuclear protein complex was significantly inhibited by addition of a 200-fold molar excess of unlabeled $2\times ACE_{core}$, but not the unlabeled $2\times mACE_{core}$.

**Table S1. A total of 847 genes contain ACE$_{core}$ in their promoter regions (-1000 bp) in *Arabidopsis*.**

| Sequence name | # of hits | Matching Positions (relative to -1000 ) | | Hit pattern |
|---|---|---|---|---|
| | | start | end | |
| **AT1G40112** | 8 | 26 | 34 | CCTAAACCC |
| | | 54 | 62 | CCTAAACCC |
| | | 68 | 76 | CCTAAACCC |
| | | 96 | 104 | CCTAAACCC |
| | | 110 | 118 | CCTAAACCC |
| | | 529 | 537 | CCTAAACCC |
| | | 585 | 593 | CCTAAACCC |
| | | 606 | 614 | CCTAAACCC |
| **AT1G40124** | 8 | 26 | 34 | CCTAAACCC |
| | | 40 | 48 | CCTAAACCC |
| | | 67 | 75 | CCTAAACCC |
| | | 115 | 123 | CCTAAACCC |
| | | 721 | 729 | CCTAAACCC |
| | | 735 | 743 | CCTAAACCC |
| | | 756 | 764 | CCTAAACCC |
| | | 770 | 778 | CCTAAACCC |
| **AT1G40129** | 8 | 292 | 300 | CCTAAACCC |
| | | 313 | 321 | CCTAAACCC |
| | | 334 | 342 | CCTAAACCC |
| | | 348 | 356 | CCTAAACCC |
| | | 760 | 768 | CCTAAACCC |
| | | 774 | 782 | CCTGAACCC |
| | | 830 | 838 | CCTAAACCC |
| | | 844 | 852 | CCTAAACCC |
| **AT1G40135** | 8 | 25 | 33 | CCTAAACCC |
| | | 39 | 47 | CCTAAACCC |
| | | 66 | 74 | CCTAAACCC |
| | | 114 | 122 | CCTAAACCC |
| | | 721 | 729 | CCTAAACCC |
| | | 735 | 743 | CCTAAACCC |
| | | 756 | 764 | CCTAAACCC |
| | | 770 | 778 | CCTAAACCC |
| **AT1G40137** | 8 | 26 | 34 | CCTAAACCC |
| | | 54 | 62 | CCTAAACCC |
| | | 68 | 76 | CCTAAACCC |
| | | 96 | 104 | CCTAAACCC |

| | | | | |
|---|---|---|---|---|
| | | 110 | 118 | CCTAAACCC |
| | | 529 | 537 | CCTAAACCC |
| | | 585 | 593 | CCTAAACCC |
| | | 606 | 614 | CCTAAACCC |
| **AT4G06511** | 8 | 264 | 272 | CCTAAACCC |
| | | 652 | 660 | CCTAAACCC |
| | | 666 | 674 | CCTAAACCC |
| | | 687 | 695 | CCTAAACCC |
| | | 714 | 722 | CCTAAACCC |
| | | 728 | 736 | CCTAAACCC |
| | | 862 | 870 | CCTAAACCC |
| | | 883 | 891 | CCTAAACCC |
| **AT1G40076** | 7 | 426 | 434 | CCTAAACCC |
| | | 440 | 448 | CCTAAACCC |
| | | 787 | 795 | CCTAAACCC |
| | | 801 | 809 | CCTAAACCC |
| | | 829 | 837 | CCTAAACCC |
| | | 857 | 865 | CCTAAACCC |
| | | 878 | 886 | CCTAAACCC |
| **AT1G40136** | 7 | 477 | 485 | CCTAAACCC |
| | | 491 | 499 | CCTAAACCC |
| | | 505 | 513 | CCTAAACCC |
| | | 519 | 527 | CCTAAACCC |
| | | 533 | 541 | CCTAAACCC |
| | | 547 | 555 | CCTAAACCC |
| | | 582 | 590 | CCTAAACCC |
| **AT2G48160** | 7 | 3 | 11 | CCTAAACCC |
| | | 17 | 25 | CCTAAACCC |
| | | 31 | 39 | CCTAAACCC |
| | | 45 | 53 | CCTAAACCC |
| | | 59 | 67 | CCTAAACCC |
| | | 73 | 81 | CCTAAACCC |
| | | 87 | 95 | CCTAAACCC |
| **AT1G40115** | 6 | 95 | 103 | CCTAAACCC |
| | | 123 | 131 | CCTAAACCC |
| | | 561 | 569 | CCTGAACCC |
| | | 575 | 583 | CCTAAACCC |
| | | 617 | 625 | CCTAAACCC |
| | | 631 | 639 | CCTAAACCC |
| **AT1G40390** | 6 | 167 | 159 | GGGTTTAGG |
| | | 263 | 255 | GGGTTTAGG |
| | | 304 | 296 | GGGTTTAGG |
| | | 690 | 682 | GGGTTTAGG |

| | | | | |
|---|---|---|---|---|
| | | 725 | 717 | GGGTTTAGG |
| | | 745 | 737 | GGGTTTAGG |
| **AT1G40083** | 5 | 144 | 152 | CCTAAACCC |
| | | 214 | 222 | CCTAAACCC |
| | | 268 | 276 | CCTAAACCC |
| | | 381 | 389 | CCTAAACCC |
| | | 431 | 439 | CCTAAACCC |
| **AT1G40084** | 5 | 591 | 583 | GGGTTTAGG |
| | | 634 | 626 | GGGTTTAGG |
| | | 754 | 746 | GGGTTTAGG |
| | | 808 | 800 | GGGTTTAGG |
| | | 871 | 863 | GGGTTTAGG |
| **AT1G40100** | 5 | 452 | 460 | CCTAAACCC |
| | | 473 | 481 | CCTAAACCC |
| | | 508 | 516 | CCTAAACCC |
| | | 918 | 926 | CCTAAACCC |
| | | 946 | 954 | CCTAAACCC |
| **AT1G40113** | 5 | 284 | 292 | CCTAAACCC |
| | | 305 | 313 | CCTAAACCC |
| | | 326 | 334 | CCTAAACCC |
| | | 340 | 348 | CCTAAACCC |
| | | 820 | 828 | CCTAAACCC |
| **AT1G40127** | 5 | 95 | 87 | GGGTTTAGG |
| | | 116 | 108 | GGGTTTAGG |
| | | 130 | 122 | GGGTTTAGG |
| | | 158 | 150 | GGGTTTAGG |
| | | 179 | 171 | GGGTTTAGG |
| **AT1G40141** | 5 | 452 | 460 | CCTAAACCC |
| | | 473 | 481 | CCTAAACCC |
| | | 508 | 516 | CCTAAACCC |
| | | 918 | 926 | CCTAAACCC |
| | | 946 | 954 | CCTAAACCC |
| **AT1G40150** | 5 | 453 | 461 | CCTAAACCC |
| | | 474 | 482 | CCTAAACCC |
| | | 509 | 517 | CCTAAACCC |
| | | 919 | 927 | CCTAAACCC |
| | | 947 | 955 | CCTAAACCC |
| **AT1G42602** | 5 | 136 | 144 | CCTAAACCC |
| | | 199 | 207 | CCTAAACCC |
| | | 390 | 398 | CCTAAACCC |
| | | 617 | 625 | CCTAAACCC |
| | | 667 | 675 | CCTAAACCC |
| **AT2G06285** | 5 | 264 | 272 | CCTAAACCC |

| | | | | |
|---|---|---|---|---|
| | | 320 | 328 | CCTAAACCC |
| | | 404 | 412 | CCTAAACCC |
| | | 474 | 482 | CCTAAACCC |
| | | 681 | 689 | CCTAAACCC |
| **AT3G42450** | 5 | 287 | 295 | CCTAAACCC |
| | | 421 | 429 | CCTAAACCC |
| | | 574 | 582 | CCTAAACCC |
| | | 602 | 610 | CCTAAACCC |
| | | 638 | 646 | CCTAAACCC |
| **AT3G42806** | 5 | 110 | 118 | CCTAAACCC |
| | | 181 | 189 | CCTAAACCC |
| | | 211 | 219 | CCTAAACCC |
| | | 234 | 242 | CCTAAACCC |
| | | 289 | 297 | CCTAAACCC |
| **AT1G40130** | 3 | 315 | 307 | GGGTTTAGG |
| | | 343 | 335 | GGGTTTAGG |
| | | 364 | 356 | GGGTTTAGG |
| **AT1G40133** | 3 | 164 | 156 | GGGTTTAGG |
| | | 192 | 184 | GGGTTTAGG |
| | | 213 | 205 | GGGTTTAGG |
| **AT4G05497** | 3 | 817 | 809 | GGGTTTAGG |
| | | 882 | 890 | CCTAGGCCC |
| | | 887 | 879 | GGGCCTAGG |
| **AT1G11740** | 2 | 156 | 148 | GGGTTTAGG |
| | | 253 | 245 | GGGTTCAGG |
| **AT1G19660** | 2 | 666 | 674 | CCTAGGCCC |
| | | 671 | 663 | GGGCCTAGG |
| **AT1G40123** | 2 | 285 | 293 | CCTAAACCC |
| | | 326 | 334 | CCTAAACCC |
| **AT1G41827** | 2 | 425 | 417 | GGGTTTAGG |
| | | 851 | 859 | CCTAAACCC |
| **AT1G42110** | 2 | 834 | 826 | GGGTTTAGG |
| | | 861 | 853 | GGGTTTAGG |
| **AT1G42816** | 2 | 360 | 352 | GGGTTTAGG |
| | | 777 | 785 | CCTAAACCC |
| **AT1G50340** | 2 | 227 | 235 | CCTAGGCCC |
| | | 232 | 224 | GGGCCTAGG |
| **AT1G50350** | 2 | 863 | 871 | CCTAGGCCC |
| | | 868 | 860 | GGGCCTAGG |
| **AT1G53520** | 2 | 27 | 35 | CCTAAACCC |
| | | 165 | 173 | CCTAAACCC |
| **AT1G64600** | 2 | 751 | 759 | CCTAGACCC |
| | | 951 | 959 | CCTAGACCC |

| | | | | |
|---|---|---|---|---|
| **AT2G07716** | 2 | 789 | 781 | GGGCTTAGG |
| | | 903 | 911 | CCTAAGCCC |
| **AT2G12510** | 2 | 167 | 175 | CCTAAACCC |
| | | 208 | 216 | CCTAAACCC |
| **AT2G15145** | 2 | 336 | 328 | GGGCCTAGG |
| | | 600 | 592 | GGGCCTAGG |
| **AT2G26320** | 2 | 443 | 435 | GGGTTTAGG |
| | | 457 | 449 | GGGTTTAGG |
| **AT3G01490** | 2 | 330 | 338 | CCTAGGCCC |
| | | 335 | 327 | GGGCCTAGG |
| **AT3G09910** | 2 | 199 | 191 | GGGCTTAGG |
| | | 240 | 232 | GGGCTTAGG |
| **AT3G33139** | 2 | 106 | 98 | GGGTTTAGG |
| | | 524 | 532 | CCTAAACCC |
| **AT3G33148** | 2 | 44 | 52 | CCTAAACCC |
| | | 62 | 70 | CCTAAACCC |
| **AT3G42780** | 2 | 171 | 179 | CCTGGACCC |
| | | 731 | 739 | CCTAAACCC |
| **AT3G44690** | 2 | 878 | 870 | GGGCCCAGG |
| | | 956 | 948 | GGGTTCAGG |
| **AT3G57350** | 2 | 142 | 150 | CCTAGACCC |
| | | 944 | 952 | CCTAAACCC |
| **AT3G63540** | 2 | 9 | 1 | GGGTTTAGG |
| | | 23 | 15 | GGGTTTAGG |
| **AT4G04440** | 2 | 291 | 283 | GGGTTTAGG |
| | | 875 | 867 | GGGTTTAGG |
| **AT4G07812** | 2 | 118 | 110 | GGGTTTAGG |
| | | 705 | 697 | GGGTTTAGG |
| **AT4G09930** | 2 | 308 | 316 | CCTAAACCC |
| | | 495 | 503 | CCTAAACCC |
| **AT4G11160** | 2 | 478 | 470 | GGGTTTAGG |
| | | 731 | 723 | GGGCCTAGG |
| **AT4G25670** | 2 | 487 | 495 | CCTAGGCCC |
| | | 492 | 484 | GGGCCTAGG |
| **AT4G25672** | 2 | 487 | 495 | CCTAGGCCC |
| | | 492 | 484 | GGGCCTAGG |
| **AT4G25680** | 2 | 434 | 442 | CCTAGGCCC |
| | | 439 | 431 | GGGCCTAGG |
| **AT4G35400** | 2 | 189 | 181 | GGGCCCAGG |
| | | 924 | 932 | CCTAAACCC |
| **AT4G39240** | 2 | 786 | 794 | CCTAAGCCC |
| | | 884 | 892 | CCTAAGCCC |
| **AT5G07340** | 2 | 730 | 722 | GGGTCTAGG |

| | | | | |
|---|---|---|---|---|
| | | 809 | 801 | GGGCCCAGG |
| **AT5G16050** | 2 | 555 | 547 | GGGTTTAGG |
| | | 741 | 733 | GGGTTCAGG |
| **AT5G29720** | 2 | 226 | 218 | GGGTTTAGG |
| | | 642 | 650 | CCTAAACCC |
| **AT5G36275** | 2 | 163 | 155 | GGGTTTAGG |
| | | 580 | 588 | CCTAAACCC |
| **AT5G40880** | 2 | 444 | 436 | GGGCTTAGG |
| | | 844 | 836 | GGGTTTAGG |
| **AT5G57200** | 2 | 256 | 264 | CCTAAACCC |
| | | 283 | 291 | CCTAAACCC |
| **AT5G65280** | 2 | 169 | 161 | GGGTTTAGG |
| | | 626 | 634 | CCTAAACCC |
| **AT5G66250** | 2 | 548 | 540 | GGGTTTAGG |
| | | 581 | 573 | GGGTCCAGG |
| **ATMG00570** | 2 | 718 | 710 | GGGCTTAGG |
| | | 832 | 840 | CCTAAGCCC |
| **AT1G01115** | 1 | 917 | 909 | GGGTTCAGG |
| **AT1G01183** | 1 | 811 | 803 | GGGTTTAGG |
| **AT1G01540** | 1 | 151 | 159 | CCTAAACCC |
| **AT1G01910** | 1 | 300 | 292 | GGGCCCAGG |
| **AT1G02690** | 1 | 916 | 924 | CCTAAGCCC |
| **AT1G02820** | 1 | 877 | 885 | CCTAAACCC |
| **AT1G03430** | 1 | 779 | 787 | CCTAAGCCC |
| **AT1G04050** | 1 | 210 | 218 | CCTGGACCC |
| **AT1G04778** | 1 | 760 | 768 | CCTAAACCC |
| **AT1G04870** | 1 | 531 | 539 | CCTAAACCC |
| **AT1G05070** | 1 | 686 | 678 | GGGTTTAGG |
| **AT1G05080** | 1 | 969 | 977 | CCTAAACCC |
| **AT1G05140** | 1 | 231 | 239 | CCTAAACCC |
| **AT1G05590** | 1 | 499 | 507 | CCTGAACCC |
| **AT1G06060** | 1 | 112 | 120 | CCTAAGCCC |
| **AT1G06160** | 1 | 181 | 173 | GGGCTTAGG |
| **AT1G06470** | 1 | 538 | 530 | GGGTCTAGG |
| **AT1G06670** | 1 | 690 | 682 | GGGCTTAGG |
| **AT1G06710** | 1 | 969 | 977 | CCTAAACCC |
| **AT1G08005** | 1 | 616 | 608 | GGGTTTAGG |
| **AT1G08160** | 1 | 16 | 24 | CCTGGGCCC |
| **AT1G08420** | 1 | 40 | 48 | CCTAAACCC |
| **AT1G08530** | 1 | 162 | 170 | CCTAGACCC |
| **AT1G09210** | 1 | 244 | 252 | CCTAAACCC |
| **AT1G09510** | 1 | 215 | 223 | CCTAGGCCC |
| **AT1G09560** | 1 | 491 | 499 | CCTAAACCC |

| | | | | |
|---|---|---|---|---|
| **AT1G09665** | 1 | 813 | 821 | CCTAAACCC |
| **AT1G09850** | 1 | 599 | 607 | CCTGAGCCC |
| **AT1G10360** | 1 | 940 | 948 | CCTAAACCC |
| **AT1G11100** | 1 | 109 | 117 | CCTAAACCC |
| **AT1G11110** | 1 | 987 | 979 | GGGTTTAGG |
| **AT1G11440** | 1 | 407 | 399 | GGGTTTAGG |
| **AT1G12211** | 1 | 980 | 972 | GGGTTTAGG |
| **AT1G12220** | 1 | 564 | 572 | CCTAAACCC |
| **AT1G12725** | 1 | 620 | 612 | GGGCTTAGG |
| **AT1G12730** | 1 | 800 | 808 | CCTAAGCCC |
| **AT1G12740** | 1 | 49 | 57 | CCTGAGCCC |
| **AT1G13090** | 1 | 783 | 791 | CCTAAACCC |
| **AT1G15200** | 1 | 352 | 360 | CCTAGACCC |
| **AT1G15360** | 1 | 64 | 56 | GGGCTTAGG |
| **AT1G15460** | 1 | 978 | 986 | CCTAAACCC |
| **AT1G15860** | 1 | 98 | 106 | CCTAAACCC |
| **AT1G15930** | 1 | 38 | 30 | GGGTTCAGG |
| **AT1G16440** | 1 | 199 | 191 | GGGTTTAGG |
| **AT1G16900** | 1 | 351 | 343 | GGGCTCAGG |
| **AT1G16916** | 1 | 971 | 963 | GGGTTTAGG |
| **AT1G17270** | 1 | 718 | 710 | GGGTTTAGG |
| **AT1G17275** | 1 | 924 | 932 | CCTAAACCC |
| **AT1G17620** | 1 | 64 | 72 | CCTGGGCCC |
| **AT1G17950** | 1 | 623 | 615 | GGGTTTAGG |
| **AT1G18335** | 1 | 337 | 345 | CCTAAACCC |
| **AT1G18590** | 1 | 260 | 252 | GGGTTTAGG |
| **AT1G18850** | 1 | 974 | 982 | CCTAAACCC |
| **AT1G19580** | 1 | 365 | 357 | GGGTTTAGG |
| **AT1G20210** | 1 | 375 | 383 | CCTAAGCCC |
| **AT1G20440** | 1 | 950 | 958 | CCTGGACCC |
| **AT1G20691** | 1 | 697 | 689 | GGGTTTAGG |
| **AT1G20693** | 1 | 983 | 991 | CCTAAACCC |
| **AT1G20696** | 1 | 949 | 957 | CCTAAACCC |
| **AT1G20960** | 1 | 876 | 868 | GGGCTTAGG |
| **AT1G21360** | 1 | 534 | 542 | CCTAAACCC |
| **AT1G21550** | 1 | 189 | 181 | GGGCTCAGG |
| **AT1G21560** | 1 | 905 | 913 | CCTGAGCCC |
| **AT1G22700** | 1 | 176 | 184 | CCTAAACCC |
| **AT1G22840** | 1 | 933 | 941 | CCTAAACCC |
| **AT1G23320** | 1 | 308 | 316 | CCTAAACCC |
| **AT1G24095** | 1 | 562 | 570 | CCTGAGCCC |
| **AT1G24110** | 1 | 108 | 100 | GGGTTTAGG |
| **AT1G24879** | 1 | 782 | 774 | GGGCTTAGG |

| | | | | |
|---|---|---|---|---|
| **AT1G25053** | 1 | 854 | 846 | GGGCTTAGG |
| **AT1G25141** | 1 | 854 | 846 | GGGCTTAGG |
| **AT1G26540** | 1 | 235 | 243 | CCTAAACCC |
| **AT1G28335** | 1 | 445 | 453 | CCTAAGCCC |
| **AT1G29410** | 1 | 815 | 807 | GGGTTTAGG |
| **AT1G29418** | 1 | 314 | 322 | CCTAAACCC |
| **AT1G29720** | 1 | 165 | 173 | CCTGAACCC |
| **AT1G30440** | 1 | 744 | 736 | GGGTTTAGG |
| **AT1G31170** | 1 | 15 | 7 | GGGTCTAGG |
| **AT1G32630** | 1 | 744 | 752 | CCTAAACCC |
| **AT1G32680** | 1 | 661 | 669 | CCTAAACCC |
| **AT1G32730** | 1 | 986 | 994 | CCTAGACCC |
| **AT1G32830** | 1 | 59 | 51 | GGGCCTAGG |
| **AT1G32980** | 1 | 344 | 336 | GGGTTTAGG |
| **AT1G33415** | 1 | 851 | 859 | CCTAAACCC |
| **AT1G33817** | 1 | 482 | 490 | CCTAAACCC |
| **AT1G34060** | 1 | 369 | 361 | GGGCTCAGG |
| **AT1G34530** | 1 | 400 | 408 | CCTAAACCC |
| **AT1G35230** | 1 | 950 | 958 | CCTAAACCC |
| **AT1G36180** | 1 | 178 | 170 | GGGTTTAGG |
| **AT1G36210** | 1 | 960 | 952 | GGGCTTAGG |
| **AT1G36595** | 1 | 121 | 113 | GGGTCTAGG |
| **AT1G36745** | 1 | 535 | 543 | CCTAAACCC |
| **AT1G36775** | 1 | 622 | 630 | CCTAAACCC |
| **AT1G36790** | 1 | 622 | 630 | CCTAAACCC |
| **AT1G37735** | 1 | 95 | 87 | GGGCTTAGG |
| **AT1G38950** | 1 | 519 | 527 | CCTGAGCCC |
| **AT1G40069** | 1 | 871 | 879 | CCTAAACCC |
| **AT1G40070** | 1 | 701 | 693 | GGGTTTAGG |
| **AT1G41726** | 1 | 201 | 193 | GGGCTCAGG |
| **AT1G41825** | 1 | 6 | 14 | CCTAAACCC |
| **AT1G41870** | 1 | 262 | 270 | CCTAAACCC |
| **AT1G42410** | 1 | 428 | 436 | CCTAAACCC |
| **AT1G43600** | 1 | 626 | 634 | CCTAGGCCC |
| **AT1G43660** | 1 | 798 | 790 | GGGCTTAGG |
| **AT1G43840** | 1 | 194 | 202 | CCTAAACCC |
| **AT1G47720** | 1 | 868 | 876 | CCTAAACCC |
| **AT1G47870** | 1 | 899 | 891 | GGGCTTAGG |
| **AT1G47980** | 1 | 963 | 971 | CCTAAACCC |
| **AT1G48250** | 1 | 924 | 932 | CCTAAACCC |
| **AT1G48400** | 1 | 632 | 640 | CCTGAACCC |
| **AT1G49170** | 1 | 404 | 396 | GGGTCTAGG |
| **AT1G49450** | 1 | 980 | 988 | CCTAAACCC |

| | | | | |
|---|---|---|---|---|
| **AT1G51150** | 1 | 754 | 762 | CCTAAGCCC |
| **AT1G51160** | 1 | 947 | 939 | GGGCTTAGG |
| **AT1G51190** | 1 | 403 | 395 | GGGCTTAGG |
| **AT1G52610** | 1 | 961 | 953 | GGGTTTAGG |
| **AT1G53530** | 1 | 35 | 43 | CCTAAACCC |
| **AT1G54690** | 1 | 991 | 999 | CCTAAACCC |
| **AT1G54700** | 1 | 400 | 392 | GGGTTTAGG |
| **AT1G55240** | 1 | 504 | 496 | GGGTTTAGG |
| **AT1G55320** | 1 | 262 | 270 | CCTGAACCC |
| **AT1G56610** | 1 | 503 | 495 | GGGTTTAGG |
| **AT1G58250** | 1 | 914 | 922 | CCTAAACCC |
| **AT1G59540** | 1 | 223 | 231 | CCTAAACCC |
| **AT1G59675** | 1 | 490 | 482 | GGGCTTAGG |
| **AT1G59680** | 1 | 894 | 902 | CCTAAGCCC |
| **AT1G60390** | 1 | 34 | 26 | GGGTTCAGG |
| **AT1G60660** | 1 | 145 | 153 | CCTAAACCC |
| **AT1G60960** | 1 | 518 | 510 | GGGTTTAGG |
| **AT1G60987** | 1 | 647 | 639 | GGGCTTAGG |
| **AT1G62110** | 1 | 939 | 947 | CCTAAACCC |
| **AT1G62820** | 1 | 894 | 886 | GGGCTTAGG |
| **AT1G62935** | 1 | 510 | 502 | GGGTTTAGG |
| **AT1G62940** | 1 | 367 | 375 | CCTAAACCC |
| **AT1G63855** | 1 | 928 | 920 | GGGCTTAGG |
| **AT1G64520** | 1 | 518 | 526 | CCTGAGCCC |
| **AT1G64580** | 1 | 407 | 399 | GGGCCTAGG |
| **AT1G64583** | 1 | 412 | 420 | CCTAAACCC |
| **AT1G64830** | 1 | 696 | 704 | CCTAAACCC |
| **AT1G64840** | 1 | 314 | 306 | GGGTTTAGG |
| **AT1G65560** | 1 | 138 | 130 | GGGCTTAGG |
| **AT1G65690** | 1 | 252 | 244 | GGGCCTAGG |
| **AT1G66380** | 1 | 866 | 874 | CCTAAGCCC |
| **AT1G66440** | 1 | 494 | 502 | CCTAAACCC |
| **AT1G66490** | 1 | 199 | 207 | CCTGAACCC |
| **AT1G67120** | 1 | 934 | 942 | CCTAAACCC |
| **AT1G68030** | 1 | 942 | 934 | GGGCCTAGG |
| **AT1G68270** | 1 | 203 | 211 | CCTGAACCC |
| **AT1G68568** | 1 | 503 | 511 | CCTAGACCC |
| **AT1G69050** | 1 | 126 | 118 | GGGTTTAGG |
| **AT1G69420** | 1 | 471 | 479 | CCTAAACCC |
| **AT1G69587** | 1 | 939 | 947 | CCTAGGCCC |
| **AT1G69800** | 1 | 891 | 883 | GGGCTCAGG |
| **AT1G70410** | 1 | 215 | 207 | GGGTCCAGG |
| **AT1G71150** | 1 | 486 | 478 | GGGTTTAGG |

| | | | | |
|---|---|---|---|---|
| **AT1G71460** | 1 | 870 | 878 | CCTAAGCCC |
| **AT1G71470** | 1 | 742 | 734 | GGGCTTAGG |
| **AT1G71910** | 1 | 352 | 360 | CCTAAACCC |
| **AT1G72260** | 1 | 258 | 266 | CCTGAACCC |
| **AT1G72840** | 1 | 184 | 192 | CCTGGACCC |
| **AT1G73920** | 1 | 656 | 648 | GGGTTTAGG |
| **AT1G74250** | 1 | 980 | 988 | CCTAAACCC |
| **AT1G74690** | 1 | 434 | 426 | GGGCTTAGG |
| **AT1G74700** | 1 | 927 | 935 | CCTAAGCCC |
| **AT1G75130** | 1 | 286 | 278 | GGGTTTAGG |
| **AT1G75310** | 1 | 472 | 480 | CCTAAACCC |
| **AT1G76600** | 1 | 105 | 113 | CCTAAACCC |
| **AT1G77340** | 1 | 806 | 814 | CCTAAACCC |
| **AT1G77350** | 1 | 771 | 763 | GGGTTTAGG |
| **AT1G78130** | 1 | 810 | 818 | CCTAAGCCC |
| **AT1G78140** | 1 | 860 | 868 | CCTAGACCC |
| **AT1G78150** | 1 | 440 | 432 | GGGTCTAGG |
| **AT1G78340** | 1 | 27 | 19 | GGGTTTAGG |
| **AT1G78350** | 1 | 924 | 932 | CCTAAACCC |
| **AT1G78760** | 1 | 517 | 509 | GGGCTTAGG |
| **AT1G78922** | 1 | 500 | 492 | GGGTTTAGG |
| **AT1G78995** | 1 | 175 | 167 | GGGCTCAGG |
| **AT1G79245** | 1 | 735 | 743 | CCTAGGCCC |
| **AT1G79820** | 1 | 430 | 438 | CCTAAGCCC |
| **AT1G79950** | 1 | 928 | 920 | GGGTTTAGG |
| **AT1G79970** | 1 | 506 | 514 | CCTAAACCC |
| **AT1G80700** | 1 | 922 | 930 | CCTAGACCC |
| **AT1G80730** | 1 | 557 | 565 | CCTAAACCC |
| **AT2G01220** | 1 | 753 | 761 | CCTAAACCC |
| **AT2G01870** | 1 | 22 | 30 | CCTGAACCC |
| **AT2G02148** | 1 | 698 | 690 | GGGTTTAGG |
| **AT2G02200** | 1 | 62 | 54 | GGGCCTAGG |
| **AT2G02230** | 1 | 88 | 80 | GGGCCTAGG |
| **AT2G02700** | 1 | 125 | 117 | GGGTTTAGG |
| **AT2G02740** | 1 | 974 | 982 | CCTAAACCC |
| **AT2G04390** | 1 | 210 | 218 | CCTAAGCCC |
| **AT2G04842** | 1 | 219 | 227 | CCTGAACCC |
| **AT2G04850** | 1 | 417 | 425 | CCTAAACCC |
| **AT2G04970** | 1 | 61 | 53 | GGGCCTAGG |
| **AT2G05680** | 1 | 249 | 257 | CCTGAACCC |
| **AT2G06440** | 1 | 67 | 59 | GGGCCTAGG |
| **AT2G06645** | 1 | 427 | 435 | CCTGAACCC |
| **AT2G06973** | 1 | 492 | 500 | CCTGAACCC |

| | | | | |
|---|---|---|---|---|
| **AT2G06980** | 1 | 802 | 794 | GGGTTCAGG |
| **AT2G07440** | 1 | 404 | 396 | GGGTCTAGG |
| **AT2G07715** | 1 | 164 | 172 | CCTAGACCC |
| **AT2G07739** | 1 | 319 | 327 | CCTAAACCC |
| **AT2G07746** | 1 | 58 | 66 | CCTGGACCC |
| **AT2G07765** | 1 | 435 | 427 | GGGTTTAGG |
| **AT2G10030** | 1 | 380 | 372 | GGGCCTAGG |
| **AT2G10256** | 1 | 293 | 301 | CCTGAACCC |
| **AT2G10921** | 1 | 684 | 676 | GGGTTTAGG |
| **AT2G11450** | 1 | 291 | 299 | CCTAAACCC |
| **AT2G11640** | 1 | 835 | 827 | GGGTCCAGG |
| **AT2G11790** | 1 | 169 | 161 | GGGTTTAGG |
| **AT2G11890** | 1 | 949 | 941 | GGGCCTAGG |
| **AT2G12560** | 1 | 435 | 443 | CCTAGACCC |
| **AT2G12670** | 1 | 75 | 83 | CCTAAACCC |
| **AT2G12780** | 1 | 240 | 248 | CCTAAACCC |
| **AT2G12815** | 1 | 260 | 252 | GGGCTCAGG |
| **AT2G12832** | 1 | 356 | 348 | GGGCTCAGG |
| **AT2G12845** | 1 | 164 | 156 | GGGCTCAGG |
| **AT2G13010** | 1 | 387 | 379 | GGGTCTAGG |
| **AT2G13160** | 1 | 435 | 443 | CCTAAACCC |
| **AT2G14140** | 1 | 60 | 52 | GGGCCTAGG |
| **AT2G14415** | 1 | 641 | 633 | GGGTTTAGG |
| **AT2G15090** | 1 | 740 | 748 | CCTAAGCCC |
| **AT2G15200** | 1 | 63 | 55 | GGGCCTAGG |
| **AT2G15240** | 1 | 981 | 989 | CCTAAACCC |
| **AT2G15370** | 1 | 269 | 261 | GGGCCTAGG |
| **AT2G15380** | 1 | 635 | 643 | CCTAGGCCC |
| **AT2G15450** | 1 | 411 | 403 | GGGCTTAGG |
| **AT2G15530** | 1 | 231 | 223 | GGGTTCAGG |
| **AT2G15800** | 1 | 780 | 788 | CCTAAACCC |
| **AT2G15930** | 1 | 464 | 472 | CCTAAACCC |
| **AT2G15940** | 1 | 602 | 594 | GGGTTTAGG |
| **AT2G16245** | 1 | 883 | 891 | CCTAAACCC |
| **AT2G16760** | 1 | 873 | 881 | CCTGGGCCC |
| **AT2G16840** | 1 | 382 | 390 | CCTAAGCCC |
| **AT2G17800** | 1 | 622 | 614 | GGGTTTAGG |
| **AT2G17970** | 1 | 533 | 541 | CCTGAACCC |
| **AT2G17990** | 1 | 312 | 320 | CCTGGACCC |
| **AT2G18180** | 1 | 67 | 59 | GGGTTTAGG |
| **AT2G19200** | 1 | 393 | 401 | CCTAAACCC |
| **AT2G20570** | 1 | 816 | 808 | GGGTTCAGG |
| **AT2G20725** | 1 | 788 | 780 | GGGTCTAGG |

| | | | | |
|---|---|---|---|---|
| **AT2G20870** | 1 | 387 | 379 | GGGTCTAGG |
| **AT2G21090** | 1 | 889 | 897 | CCTAAGCCC |
| **AT2G21360** | 1 | 368 | 376 | CCTAAACCC |
| **AT2G21630** | 1 | 653 | 661 | CCTAAACCC |
| **AT2G22410** | 1 | 489 | 497 | CCTAAACCC |
| **AT2G22980** | 1 | 466 | 458 | GGGTCTAGG |
| **AT2G23160** | 1 | 179 | 187 | CCTAAACCC |
| **AT2G23510** | 1 | 203 | 211 | CCTAAACCC |
| **AT2G24540** | 1 | 444 | 452 | CCTAAACCC |
| **AT2G24693** | 1 | 509 | 517 | CCTAGACCC |
| **AT2G24860** | 1 | 929 | 921 | GGGCCCAGG |
| **AT2G25070** | 1 | 287 | 279 | GGGTCTAGG |
| **AT2G25330** | 1 | 915 | 923 | CCTAAGCCC |
| **AT2G26350** | 1 | 172 | 180 | CCTAAGCCC |
| **AT2G26430** | 1 | 914 | 922 | CCTAAGCCC |
| **AT2G26690** | 1 | 321 | 313 | GGGTTTAGG |
| **AT2G26790** | 1 | 943 | 935 | GGGTTTAGG |
| **AT2G27935** | 1 | 482 | 474 | GGGTTTAGG |
| **AT2G28360** | 1 | 372 | 380 | CCTAAGCCC |
| **AT2G28370** | 1 | 145 | 137 | GGGCTTAGG |
| **AT2G29320** | 1 | 791 | 783 | GGGTTTAGG |
| **AT2G29420** | 1 | 785 | 777 | GGGTTCAGG |
| **AT2G29430** | 1 | 562 | 570 | CCTGAACCC |
| **AT2G29480** | 1 | 396 | 388 | GGGTTTAGG |
| **AT2G29990** | 1 | 282 | 290 | CCTAAGCCC |
| **AT2G30080** | 1 | 746 | 738 | GGGCTCAGG |
| **AT2G31130** | 1 | 900 | 908 | CCTGGGCCC |
| **AT2G31650** | 1 | 867 | 875 | CCTAAACCC |
| **AT2G31820** | 1 | 960 | 952 | GGGTTTAGG |
| **AT2G32870** | 1 | 956 | 964 | CCTAAACCC |
| **AT2G33020** | 1 | 554 | 562 | CCTAGACCC |
| **AT2G33060** | 1 | 304 | 312 | CCTAAACCC |
| **AT2G33220** | 1 | 865 | 857 | GGGTTTAGG |
| **AT2G33510** | 1 | 703 | 711 | CCTAAACCC |
| **AT2G34130** | 1 | 195 | 203 | CCTAAACCC |
| **AT2G34820** | 1 | 125 | 133 | CCTAGGCCC |
| **AT2G34880** | 1 | 338 | 330 | GGGCTTAGG |
| **AT2G35744** | 1 | 565 | 557 | GGGTTTAGG |
| **AT2G35747** | 1 | 686 | 678 | GGGTTTAGG |
| **AT2G35765** | 1 | 66 | 58 | GGGTTTAGG |
| **AT2G36485** | 1 | 143 | 151 | CCTAAACCC |
| **AT2G36620** | 1 | 993 | 985 | GGGCTCAGG |
| **AT2G37030** | 1 | 550 | 558 | CCTGGACCC |

| | | | | |
|---|---|---|---|---|
| **AT2G38195** | 1 | 776 | 768 | GGGCTTAGG |
| **AT2G39270** | 1 | 628 | 620 | GGGTTCAGG |
| **AT2G39440** | 1 | 11 | 19 | CCTAAACCC |
| **AT2G40590** | 1 | 918 | 926 | CCTAAGCCC |
| **AT2G40710** | 1 | 271 | 263 | GGGTTTAGG |
| **AT2G40780** | 1 | 702 | 710 | CCTGAACCC |
| **AT2G41473** | 1 | 827 | 835 | CCTAAACCC |
| **AT2G41475** | 1 | 333 | 325 | GGGTTTAGG |
| **AT2G42480** | 1 | 548 | 540 | GGGTTTAGG |
| **AT2G42485** | 1 | 851 | 859 | CCTAAACCC |
| **AT2G42690** | 1 | 540 | 532 | GGGTCTAGG |
| **AT2G42700** | 1 | 905 | 913 | CCTAGACCC |
| **AT2G43770** | 1 | 381 | 389 | CCTAGGCCC |
| **AT2G43790** | 1 | 280 | 288 | CCTAAACCC |
| **AT2G43830** | 1 | 807 | 815 | CCTAAACCC |
| **AT2G43840** | 1 | 45 | 37 | GGGTTTAGG |
| **AT2G45410** | 1 | 525 | 517 | GGGTTCAGG |
| **AT2G45460** | 1 | 569 | 577 | CCTGGGCCC |
| **AT2G45790** | 1 | 33 | 41 | CCTGAGCCC |
| **AT2G45940** | 1 | 90 | 98 | CCTAAACCC |
| **AT2G46870** | 1 | 699 | 707 | CCTAAGCCC |
| **AT2G47810** | 1 | 797 | 789 | GGGTTTAGG |
| **AT2G48070** | 1 | 30 | 38 | CCTAAACCC |
| **AT3G01060** | 1 | 44 | 52 | CCTGAGCCC |
| **AT3G01540** | 1 | 886 | 878 | GGGCCTAGG |
| **AT3G01790** | 1 | 184 | 192 | CCTAAACCC |
| **AT3G02130** | 1 | 961 | 953 | GGGTTTAGG |
| **AT3G02560** | 1 | 142 | 134 | GGGCTTAGG |
| **AT3G03370** | 1 | 479 | 471 | GGGTTTAGG |
| **AT3G03770** | 1 | 391 | 399 | CCTAAACCC |
| **AT3G03960** | 1 | 127 | 135 | CCTAAACCC |
| **AT3G03970** | 1 | 946 | 954 | CCTAAACCC |
| **AT3G04870** | 1 | 955 | 947 | GGGTTTAGG |
| **AT3G04945** | 1 | 562 | 554 | GGGTTTAGG |
| **AT3G05060** | 1 | 908 | 916 | CCTAAACCC |
| **AT3G05070** | 1 | 912 | 904 | GGGTTTAGG |
| **AT3G05220** | 1 | 182 | 190 | CCTAAACCC |
| **AT3G06035** | 1 | 294 | 302 | CCTAGACCC |
| **AT3G06210** | 1 | 622 | 614 | GGGTTTAGG |
| **AT3G06280** | 1 | 936 | 944 | CCTAAACCC |
| **AT3G06580** | 1 | 384 | 392 | CCTAAACCC |
| **AT3G06810** | 1 | 257 | 249 | GGGCCCAGG |
| **AT3G06870** | 1 | 570 | 578 | CCTAAACCC |

| | | | | |
|---|---|---|---|---|
| **AT3G07215** | 1 | 380 | 388 | CCTGGACCC |
| **AT3G07290** | 1 | 930 | 938 | CCTAAACCC |
| **AT3G07710** | 1 | 299 | 307 | CCTGAACCC |
| **AT3G07850** | 1 | 649 | 641 | GGGTTTAGG |
| **AT3G08000** | 1 | 827 | 819 | GGGTTTAGG |
| **AT3G09032** | 1 | 516 | 508 | GGGTTTAGG |
| **AT3G09035** | 1 | 763 | 771 | CCTAAACCC |
| **AT3G09720** | 1 | 350 | 358 | CCTAAACCC |
| **AT3G09760** | 1 | 647 | 655 | CCTAAGCCC |
| **AT3G10160** | 1 | 819 | 811 | GGGCTTAGG |
| **AT3G10185** | 1 | 127 | 135 | CCTAAGCCC |
| **AT3G10670** | 1 | 778 | 770 | GGGTTTAGG |
| **AT3G10680** | 1 | 838 | 846 | CCTAAACCC |
| **AT3G11010** | 1 | 182 | 190 | CCTAAACCC |
| **AT3G11020** | 1 | 148 | 140 | GGGTTTAGG |
| **AT3G11395** | 1 | 103 | 111 | CCTAGACCC |
| **AT3G11570** | 1 | 70 | 62 | GGGTTTAGG |
| **AT3G11740** | 1 | 139 | 131 | GGGTTTAGG |
| **AT3G11820** | 1 | 709 | 701 | GGGTTTAGG |
| **AT3G11825** | 1 | 680 | 688 | CCTAAACCC |
| **AT3G12740** | 1 | 39 | 31 | GGGTTTAGG |
| **AT3G13150** | 1 | 973 | 981 | CCTAAACCC |
| **AT3G13226** | 1 | 407 | 399 | GGGTTTAGG |
| **AT3G13490** | 1 | 934 | 926 | GGGTTTAGG |
| **AT3G13500** | 1 | 419 | 427 | CCTAAACCC |
| **AT3G13770** | 1 | 109 | 117 | CCTAGACCC |
| **AT3G13965** | 1 | 299 | 291 | GGGCTTAGG |
| **AT3G14070** | 1 | 922 | 930 | CCTAGGCCC |
| **AT3G14290** | 1 | 845 | 837 | GGGTTTAGG |
| **AT3G14362** | 1 | 459 | 467 | CCTAAACCC |
| **AT3G14910** | 1 | 820 | 812 | GGGTTCAGG |
| **AT3G15280** | 1 | 588 | 596 | CCTGAACCC |
| **AT3G15357** | 1 | 978 | 986 | CCTAAACCC |
| **AT3G15540** | 1 | 193 | 201 | CCTGAACCC |
| **AT3G15740** | 1 | 954 | 962 | CCTAAACCC |
| **AT3G15750** | 1 | 523 | 515 | GGGTTTAGG |
| **AT3G15909** | 1 | 937 | 929 | GGGTTTAGG |
| **AT3G15910** | 1 | 479 | 471 | GGGTTTAGG |
| **AT3G16500** | 1 | 802 | 794 | GGGTTTAGG |
| **AT3G16740** | 1 | 45 | 53 | CCTAAACCC |
| **AT3G16760** | 1 | 39 | 47 | CCTGAACCC |
| **AT3G17140** | 1 | 215 | 207 | GGGCTTAGG |
| **AT3G17609** | 1 | 739 | 747 | CCTAAACCC |

| | | | | |
|---|---|---|---|---|
| **AT3G18217** | 1 | 327 | 319 | GGGTTTAGG |
| **AT3G18730** | 1 | 501 | 509 | CCTAAACCC |
| **AT3G18773** | 1 | 299 | 291 | GGGCTCAGG |
| **AT3G19515** | 1 | 919 | 927 | CCTGAACCC |
| **AT3G19615** | 1 | 107 | 115 | CCTAAACCC |
| **AT3G19740** | 1 | 110 | 118 | CCTGAACCC |
| **AT3G19980** | 1 | 116 | 124 | CCTAAACCC |
| **AT3G20015** | 1 | 797 | 789 | GGGTTTAGG |
| **AT3G20070** | 1 | 284 | 276 | GGGTTTAGG |
| **AT3G20080** | 1 | 767 | 775 | CCTAAACCC |
| **AT3G20200** | 1 | 46 | 38 | GGGTTTAGG |
| **AT3G20420** | 1 | 800 | 792 | GGGTTTAGG |
| **AT3G20690** | 1 | 723 | 715 | GGGTTTAGG |
| **AT3G20960** | 1 | 267 | 275 | CCTAAACCC |
| **AT3G21100** | 1 | 747 | 755 | CCTAAACCC |
| **AT3G21800** | 1 | 534 | 526 | GGGTTTAGG |
| **AT3G21805** | 1 | 791 | 799 | CCTAAACCC |
| **AT3G22560** | 1 | 706 | 698 | GGGTTTAGG |
| **AT3G22950** | 1 | 929 | 937 | CCTAAGCCC |
| **AT3G22955** | 1 | 660 | 652 | GGGCTTAGG |
| **AT3G23390** | 1 | 129 | 121 | GGGTTCAGG |
| **AT3G23700** | 1 | 434 | 442 | CCTGAGCCC |
| **AT3G23720** | 1 | 738 | 746 | CCTAAACCC |
| **AT3G24080** | 1 | 459 | 451 | GGGCTTAGG |
| **AT3G24350** | 1 | 324 | 316 | GGGTTTAGG |
| **AT3G24506** | 1 | 687 | 679 | GGGTTCAGG |
| **AT3G24508** | 1 | 488 | 496 | CCTGAACCC |
| **AT3G24530** | 1 | 882 | 874 | GGGTTTAGG |
| **AT3G24540** | 1 | 305 | 313 | CCTAAACCC |
| **AT3G25990** | 1 | 35 | 27 | GGGCTCAGG |
| **AT3G26540** | 1 | 923 | 931 | CCTAAACCC |
| **AT3G26600** | 1 | 429 | 437 | CCTAAGCCC |
| **AT3G26614** | 1 | 483 | 491 | CCTAAGCCC |
| **AT3G26618** | 1 | 485 | 477 | GGGCTTAGG |
| **AT3G26860** | 1 | 589 | 581 | GGGTCTAGG |
| **AT3G28150** | 1 | 325 | 333 | CCTAAGCCC |
| **AT3G28193** | 1 | 842 | 834 | GGGTTCAGG |
| **AT3G28270** | 1 | 46 | 38 | GGGTTTAGG |
| **AT3G28290** | 1 | 467 | 475 | CCTAAACCC |
| **AT3G28300** | 1 | 467 | 475 | CCTAAACCC |
| **AT3G28350** | 1 | 907 | 915 | CCTAAACCC |
| **AT3G29105** | 1 | 587 | 579 | GGGTTTAGG |
| **AT3G29150** | 1 | 448 | 440 | GGGTCCAGG |

| | | | | |
|---|---|---|---|---|
| **AT3G29680** | 1 | 742 | 734 | GGGTTTAGG |
| **AT3G29730** | 1 | 296 | 304 | CCTAAACCC |
| **AT3G29796** | 1 | 857 | 865 | CCTAGACCC |
| **AT3G30396** | 1 | 370 | 378 | CCTAAACCC |
| **AT3G30450** | 1 | 62 | 54 | GGGTCTAGG |
| **AT3G30718** | 1 | 139 | 131 | GGGTTTAGG |
| **AT3G30722** | 1 | 461 | 453 | GGGCCTAGG |
| **AT3G30730** | 1 | 485 | 493 | CCTGAACCC |
| **AT3G30846** | 1 | 605 | 597 | GGGCCCAGG |
| **AT3G31374** | 1 | 531 | 523 | GGGTTTAGG |
| **AT3G31375** | 1 | 537 | 545 | CCTAAACCC |
| **AT3G31400** | 1 | 675 | 683 | CCTAAACCC |
| **AT3G32031** | 1 | 104 | 112 | CCTAAACCC |
| **AT3G32116** | 1 | 662 | 670 | CCTAAACCC |
| **AT3G32391** | 1 | 45 | 37 | GGGCCTAGG |
| **AT3G32910** | 1 | 60 | 52 | GGGCCTAGG |
| **AT3G32975** | 1 | 682 | 674 | GGGTTTAGG |
| **AT3G33230** | 1 | 655 | 663 | CCTAAGCCC |
| **AT3G35003** | 1 | 754 | 746 | GGGCTTAGG |
| **AT3G42090** | 1 | 220 | 228 | CCTAAGCCC |
| **AT3G42258** | 1 | 185 | 177 | GGGTCTAGG |
| **AT3G42300** | 1 | 224 | 216 | GGGTTTAGG |
| **AT3G42472** | 1 | 538 | 530 | GGGTTTAGG |
| **AT3G42720** | 1 | 148 | 156 | CCTAAACCC |
| **AT3G42820** | 1 | 652 | 644 | GGGTCTAGG |
| **AT3G43170** | 1 | 318 | 310 | GGGTTCAGG |
| **AT3G43546** | 1 | 753 | 745 | GGGCTTAGG |
| **AT3G44180** | 1 | 137 | 129 | GGGTTTAGG |
| **AT3G45280** | 1 | 565 | 573 | CCTAGGCCC |
| **AT3G45285** | 1 | 211 | 203 | GGGCCTAGG |
| **AT3G45970** | 1 | 165 | 157 | GGGTTTAGG |
| **AT3G46310** | 1 | 220 | 228 | CCTAAGCCC |
| **AT3G46730** | 1 | 222 | 230 | CCTAAACCC |
| **AT3G47080** | 1 | 30 | 38 | CCTAAACCC |
| **AT3G47720** | 1 | 73 | 65 | GGGCTTAGG |
| **AT3G48460** | 1 | 608 | 600 | GGGTTTAGG |
| **AT3G48720** | 1 | 912 | 920 | CCTAAACCC |
| **AT3G48810** | 1 | 117 | 125 | CCTAAACCC |
| **AT3G48980** | 1 | 89 | 97 | CCTAAACCC |
| **AT3G49530** | 1 | 159 | 151 | GGGTTTAGG |
| **AT3G49601** | 1 | 948 | 956 | CCTAAGCCC |
| **AT3G52900** | 1 | 467 | 475 | CCTAAACCC |
| **AT3G53410** | 1 | 553 | 561 | CCTGAACCC |

| | | | | |
|---|---|---|---|---|
| **AT3G53640** | 1 | 815 | 823 | CCTGGGCCC |
| **AT3G53650** | 1 | 764 | 756 | GGGCCCAGG |
| **AT3G53900** | 1 | 253 | 245 | GGGCTTAGG |
| **AT3G54085** | 1 | 14 | 22 | CCTAAACCC |
| **AT3G54540** | 1 | 589 | 581 | GGGTTCAGG |
| **AT3G54680** | 1 | 348 | 340 | GGGTTTAGG |
| **AT3G55290** | 1 | 852 | 860 | CCTAGACCC |
| **AT3G55480** | 1 | 864 | 856 | GGGTTTAGG |
| **AT3G55485** | 1 | 952 | 960 | CCTAAACCC |
| **AT3G55512** | 1 | 522 | 530 | CCTAAACCC |
| **AT3G55573** | 1 | 537 | 529 | GGGTTTAGG |
| **AT3G55580** | 1 | 193 | 201 | CCTAAACCC |
| **AT3G57360** | 1 | 823 | 815 | GGGTTTAGG |
| **AT3G57810** | 1 | 337 | 329 | GGGCCCAGG |
| **AT3G57840** | 1 | 623 | 615 | GGGTTTAGG |
| **AT3G57854** | 1 | 630 | 622 | GGGTTTAGG |
| **AT3G57930** | 1 | 370 | 362 | GGGTTTAGG |
| **AT3G58470** | 1 | 469 | 461 | GGGTCTAGG |
| **AT3G58530** | 1 | 798 | 790 | GGGTTTAGG |
| **AT3G58600** | 1 | 782 | 774 | GGGCCTAGG |
| **AT3G58610** | 1 | 954 | 962 | CCTAGGCCC |
| **AT3G59520** | 1 | 894 | 902 | CCTAGGCCC |
| **AT3G59700** | 1 | 217 | 209 | GGGTCCAGG |
| **AT3G60972** | 1 | 781 | 789 | CCTGAACCC |
| **AT3G61740** | 1 | 399 | 407 | CCTAGACCC |
| **AT3G61962** | 1 | 340 | 332 | GGGTCTAGG |
| **AT3G61970** | 1 | 534 | 542 | CCTAAGCCC |
| **AT3G62285** | 1 | 2 | 10 | CCTAAGCCC |
| **AT3G62740** | 1 | 610 | 602 | GGGCTTAGG |
| **AT3G62900** | 1 | 959 | 951 | GGGCCTAGG |
| **AT3G63020** | 1 | 103 | 111 | CCTAAACCC |
| **AT3G63130** | 1 | 410 | 418 | CCTAAACCC |
| **AT3G63390** | 1 | 677 | 685 | CCTAAACCC |
| **AT3G63445** | 1 | 21 | 13 | GGGTTCAGG |
| **AT4G00710** | 1 | 643 | 651 | CCTAAACCC |
| **AT4G01000** | 1 | 240 | 248 | CCTAAACCC |
| **AT4G01593** | 1 | 662 | 654 | GGGTTCAGG |
| **AT4G01800** | 1 | 513 | 521 | CCTGAGCCC |
| **AT4G01940** | 1 | 426 | 418 | GGGCCTAGG |
| **AT4G02540** | 1 | 857 | 865 | CCTAAACCC |
| **AT4G02860** | 1 | 100 | 108 | CCTAAACCC |
| **AT4G02880** | 1 | 837 | 845 | CCTAAACCC |
| **AT4G03305** | 1 | 678 | 670 | GGGTTTAGG |

| | | | | |
|---|---|---|---|---|
| **AT4G03370** | 1 | 490 | 482 | GGGTTTAGG |
| **AT4G03405** | 1 | 676 | 684 | CCTAAACCC |
| **AT4G03560** | 1 | 453 | 445 | GGGTTTAGG |
| **AT4G03635** | 1 | 873 | 881 | CCTAGGCCC |
| **AT4G04010** | 1 | 163 | 155 | GGGTTCAGG |
| **AT4G04020** | 1 | 91 | 99 | CCTGAACCC |
| **AT4G04221** | 1 | 479 | 487 | CCTAAACCC |
| **AT4G04430** | 1 | 428 | 436 | CCTAAACCC |
| **AT4G04550** | 1 | 832 | 840 | CCTAAGCCC |
| **AT4G04940** | 1 | 992 | 1000 | CCTAAACCC |
| **AT4G05410** | 1 | 146 | 154 | CCTAAACCC |
| **AT4G05450** | 1 | 561 | 553 | GGGCTCAGG |
| **AT4G05502** | 1 | 739 | 731 | GGGTTTAGG |
| **AT4G05505** | 1 | 831 | 839 | CCTAAACCC |
| **AT4G05553** | 1 | 117 | 125 | CCTAAACCC |
| **AT4G06503** | 1 | 242 | 250 | CCTAAACCC |
| **AT4G06504** | 1 | 928 | 920 | GGGTTTAGG |
| **AT4G06551** | 1 | 293 | 285 | GGGTTTAGG |
| **AT4G07733** | 1 | 222 | 214 | GGGTTTAGG |
| **AT4G07970** | 1 | 184 | 176 | GGGCCTAGG |
| **AT4G08013** | 1 | 887 | 895 | CCTAAACCC |
| **AT4G08098** | 1 | 915 | 923 | CCTAAGCCC |
| **AT4G08134** | 1 | 45 | 37 | GGGTTTAGG |
| **AT4G08351** | 1 | 563 | 555 | GGGTTCAGG |
| **AT4G08691** | 1 | 11 | 3 | GGGTTTAGG |
| **AT4G08876** | 1 | 410 | 402 | GGGCCTAGG |
| **AT4G08991** | 1 | 659 | 651 | GGGTTTAGG |
| **AT4G09360** | 1 | 273 | 281 | CCTAAACCC |
| **AT4G09647** | 1 | 171 | 163 | GGGTTTAGG |
| **AT4G10510** | 1 | 356 | 348 | GGGTTTAGG |
| **AT4G11640** | 1 | 644 | 652 | CCTAAACCC |
| **AT4G11670** | 1 | 961 | 969 | CCTAAACCC |
| **AT4G12040** | 1 | 351 | 359 | CCTGAACCC |
| **AT4G12700** | 1 | 479 | 471 | GGGTTTAGG |
| **AT4G13000** | 1 | 90 | 82 | GGGTTTAGG |
| **AT4G13010** | 1 | 618 | 626 | CCTAAACCC |
| **AT4G13750** | 1 | 844 | 852 | CCTAAACCC |
| **AT4G15030** | 1 | 223 | 215 | GGGTTCAGG |
| **AT4G15393** | 1 | 138 | 146 | CCTAAACCC |
| **AT4G15570** | 1 | 986 | 994 | CCTAAACCC |
| **AT4G16045** | 1 | 308 | 300 | GGGTTTAGG |
| **AT4G16447** | 1 | 264 | 272 | CCTAAACCC |
| **AT4G16490** | 1 | 774 | 782 | CCTGGACCC |

| | | | | |
|---|---|---|---|---|
| **AT4G17210** | 1 | 560 | 552 | GGGTTTAGG |
| **AT4G17250** | 1 | 510 | 518 | CCTAGACCC |
| **AT4G17330** | 1 | 398 | 406 | CCTAAGCCC |
| **AT4G17410** | 1 | 372 | 364 | GGGTTCAGG |
| **AT4G18140** | 1 | 837 | 845 | CCTAAGCCC |
| **AT4G18150** | 1 | 392 | 384 | GGGCTTAGG |
| **AT4G18510** | 1 | 791 | 799 | CCTAGGCCC |
| **AT4G20240** | 1 | 936 | 944 | CCTAAACCC |
| **AT4G20365** | 1 | 885 | 877 | GGGTTTAGG |
| **AT4G21105** | 1 | 474 | 482 | CCTAAACCC |
| **AT4G21970** | 1 | 280 | 288 | CCTGGACCC |
| **AT4G22190** | 1 | 244 | 236 | GGGTCCAGG |
| **AT4G22380** | 1 | 523 | 531 | CCTAAACCC |
| **AT4G23630** | 1 | 660 | 652 | GGGTTTAGG |
| **AT4G23780** | 1 | 883 | 875 | GGGTTTAGG |
| **AT4G23790** | 1 | 78 | 86 | CCTAAACCC |
| **AT4G23960** | 1 | 953 | 945 | GGGTTTAGG |
| **AT4G24265** | 1 | 333 | 341 | CCTAAACCC |
| **AT4G25235** | 1 | 66 | 74 | CCTAAACCC |
| **AT4G25260** | 1 | 976 | 984 | CCTGGACCC |
| **AT4G25730** | 1 | 968 | 976 | CCTAAACCC |
| **AT4G25990** | 1 | 862 | 854 | GGGTTTAGG |
| **AT4G26130** | 1 | 595 | 587 | GGGCTCAGG |
| **AT4G26660** | 1 | 658 | 650 | GGGTCCAGG |
| **AT4G26795** | 1 | 759 | 767 | CCTAAACCC |
| **AT4G27270** | 1 | 341 | 333 | GGGTTTAGG |
| **AT4G27340** | 1 | 822 | 830 | CCTAAACCC |
| **AT4G27580** | 1 | 330 | 322 | GGGTTTAGG |
| **AT4G27800** | 1 | 931 | 939 | CCTAAGCCC |
| **AT4G28520** | 1 | 263 | 255 | GGGTTTAGG |
| **AT4G29310** | 1 | 919 | 927 | CCTGGGCCC |
| **AT4G29920** | 1 | 977 | 969 | GGGTTTAGG |
| **AT4G29930** | 1 | 407 | 415 | CCTGGACCC |
| **AT4G30500** | 1 | 830 | 838 | CCTAAACCC |
| **AT4G30820** | 1 | 922 | 930 | CCTAAGCCC |
| **AT4G31660** | 1 | 188 | 180 | GGGTTCAGG |
| **AT4G33905** | 1 | 984 | 992 | CCTGAACCC |
| **AT4G33940** | 1 | 223 | 231 | CCTGAACCC |
| **AT4G34350** | 1 | 151 | 159 | CCTAAACCC |
| **AT4G34500** | 1 | 559 | 551 | GGGTTTAGG |
| **AT4G34510** | 1 | 601 | 609 | CCTAAACCC |
| **AT4G34610** | 1 | 752 | 744 | GGGTTTAGG |
| **AT4G35080** | 1 | 744 | 752 | CCTAAACCC |

| | | | | |
|---|---|---|---|---|
| **AT4G35519** | 1 | 922 | 930 | CCTAGACCC |
| **AT4G35520** | 1 | 962 | 954 | GGGTCTAGG |
| **AT4G35640** | 1 | 684 | 676 | GGGTCTAGG |
| **AT4G35850** | 1 | 949 | 957 | CCTAAACCC |
| **AT4G36350** | 1 | 357 | 349 | GGGTTCAGG |
| **AT4G36390** | 1 | 541 | 549 | CCTAAACCC |
| **AT4G36480** | 1 | 909 | 901 | GGGCCCAGG |
| **AT4G36610** | 1 | 371 | 379 | CCTAAGCCC |
| **AT4G36850** | 1 | 500 | 492 | GGGCTTAGG |
| **AT4G37095** | 1 | 854 | 862 | CCTAAACCC |
| **AT4G37432** | 1 | 894 | 886 | GGGCCTAGG |
| **AT4G37910** | 1 | 832 | 824 | GGGTCCAGG |
| **AT4G38400** | 1 | 332 | 340 | CCTAAACCC |
| **AT4G38401** | 1 | 477 | 485 | CCTAAACCC |
| **AT4G38410** | 1 | 470 | 462 | GGGTTTAGG |
| **AT4G39090** | 1 | 310 | 302 | GGGTTTAGG |
| **AT4G39680** | 1 | 757 | 749 | GGGTTCAGG |
| **AT4G39795** | 1 | 312 | 320 | CCTAGGCCC |
| **AT5G01520** | 1 | 524 | 532 | CCTAAACCC |
| **AT5G01580** | 1 | 662 | 654 | GGGTTCAGG |
| **AT5G01650** | 1 | 793 | 785 | GGGTCTAGG |
| **AT5G01820** | 1 | 726 | 734 | CCTAAACCC |
| **AT5G02050** | 1 | 537 | 545 | CCTAAACCC |
| **AT5G02230** | 1 | 83 | 75 | GGGTCTAGG |
| **AT5G02870** | 1 | 769 | 777 | CCTAAACCC |
| **AT5G02960** | 1 | 140 | 148 | CCTAAACCC |
| **AT5G03460** | 1 | 665 | 673 | CCTAAACCC |
| **AT5G03630** | 1 | 469 | 461 | GGGTTTAGG |
| **AT5G03690** | 1 | 66 | 58 | GGGTTTAGG |
| **AT5G03910** | 1 | 988 | 980 | GGGTTTAGG |
| **AT5G03920** | 1 | 40 | 48 | CCTAAACCC |
| **AT5G03970** | 1 | 805 | 813 | CCTAAACCC |
| **AT5G03990** | 1 | 347 | 339 | GGGTTTAGG |
| **AT5G04130** | 1 | 964 | 956 | GGGTTTAGG |
| **AT5G04280** | 1 | 927 | 919 | GGGCCTAGG |
| **AT5G04350** | 1 | 351 | 343 | GGGCCTAGG |
| **AT5G04895** | 1 | 862 | 870 | CCTAAACCC |
| **AT5G05050** | 1 | 888 | 896 | CCTGGACCC |
| **AT5G05700** | 1 | 33 | 25 | GGGTTTAGG |
| **AT5G05720** | 1 | 829 | 837 | CCTAAACCC |
| **AT5G05890** | 1 | 912 | 904 | GGGCCTAGG |
| **AT5G06120** | 1 | 381 | 389 | CCTAAACCC |
| **AT5G06190** | 1 | 565 | 557 | GGGTTTAGG |

| | | | | |
|---|---|---|---|---|
| **AT5G06200** | 1 | 886 | 894 | CCTAAACCC |
| **AT5G06410** | 1 | 732 | 740 | CCTAAACCC |
| **AT5G07050** | 1 | 377 | 385 | CCTAAACCC |
| **AT5G07370** | 1 | 692 | 684 | GGGTTTAGG |
| **AT5G07920** | 1 | 808 | 816 | CCTGGGCCC |
| **AT5G08170** | 1 | 745 | 737 | GGGTTTAGG |
| **AT5G08310** | 1 | 951 | 943 | GGGTTTAGG |
| **AT5G08440** | 1 | 205 | 213 | CCTGGACCC |
| **AT5G08600** | 1 | 562 | 554 | GGGCTCAGG |
| **AT5G10670** | 1 | 877 | 885 | CCTAAACCC |
| **AT5G10680** | 1 | 379 | 371 | GGGTTTAGG |
| **AT5G10980** | 1 | 678 | 686 | CCTGAACCC |
| **AT5G11412** | 1 | 364 | 372 | CCTAAACCC |
| **AT5G11470** | 1 | 11 | 19 | CCTAAACCC |
| **AT5G11820** | 1 | 649 | 641 | GGGTCTAGG |
| **AT5G12210** | 1 | 13 | 21 | CCTAAACCC |
| **AT5G12323** | 1 | 58 | 50 | GGGTTTAGG |
| **AT5G13030** | 1 | 229 | 237 | CCTAAACCC |
| **AT5G13240** | 1 | 319 | 327 | CCTAAACCC |
| **AT5G15254** | 1 | 365 | 357 | GGGCTTAGG |
| **AT5G15600** | 1 | 523 | 531 | CCTAAGCCC |
| **AT5G15720** | 1 | 519 | 527 | CCTAAACCC |
| **AT5G15725** | 1 | 260 | 252 | GGGTTTAGG |
| **AT5G16060** | 1 | 961 | 969 | CCTGAACCC |
| **AT5G16250** | 1 | 241 | 233 | GGGCCCAGG |
| **AT5G16336** | 1 | 823 | 831 | CCTAAACCC |
| **AT5G16950** | 1 | 90 | 82 | GGGTTTAGG |
| **AT5G17233** | 1 | 945 | 937 | GGGTTTAGG |
| **AT5G17660** | 1 | 434 | 442 | CCTAAACCC |
| **AT5G17710** | 1 | 352 | 360 | CCTAAGCCC |
| **AT5G19830** | 1 | 950 | 958 | CCTAAACCC |
| **AT5G20290** | 1 | 171 | 163 | GGGTTCAGG |
| **AT5G20650** | 1 | 435 | 427 | GGGTTTAGG |
| **AT5G22310** | 1 | 573 | 581 | CCTAAACCC |
| **AT5G22500** | 1 | 609 | 617 | CCTAAACCC |
| **AT5G23110** | 1 | 255 | 263 | CCTAAACCC |
| **AT5G23411** | 1 | 779 | 787 | CCTAGACCC |
| **AT5G24160** | 1 | 229 | 237 | CCTAAACCC |
| **AT5G25210** | 1 | 97 | 105 | CCTGAACCC |
| **AT5G25425** | 1 | 986 | 978 | GGGCTCAGG |
| **AT5G26780** | 1 | 734 | 726 | GGGTTTAGG |
| **AT5G26800** | 1 | 853 | 861 | CCTAAACCC |
| **AT5G26810** | 1 | 107 | 115 | CCTAAACCC |

| | | | | |
|---|---|---|---|---|
| **AT5G28470** | 1 | 802 | 810 | CCTAAGCCC |
| **AT5G28495** | 1 | 195 | 187 | GGGTTTAGG |
| **AT5G28675** | 1 | 436 | 444 | CCTGAACCC |
| **AT5G28850** | 1 | 862 | 870 | CCTAAACCC |
| **AT5G28900** | 1 | 913 | 921 | CCTAAACCC |
| **AT5G29058** | 1 | 348 | 356 | CCTAAACCC |
| **AT5G29408** | 1 | 54 | 62 | CCTAAACCC |
| **AT5G29762** | 1 | 626 | 634 | CCTAAACCC |
| **AT5G31909** | 1 | 351 | 343 | GGGTCTAGG |
| **AT5G32022** | 1 | 394 | 402 | CCTAAACCC |
| **AT5G32580** | 1 | 822 | 830 | CCTGAGCCC |
| **AT5G32900** | 1 | 919 | 927 | CCTAGGCCC |
| **AT5G32950** | 1 | 423 | 431 | CCTAAACCC |
| **AT5G33381** | 1 | 227 | 219 | GGGTTTAGG |
| **AT5G33420** | 1 | 33 | 41 | CCTAAACCC |
| **AT5G33806** | 1 | 831 | 839 | CCTGAGCCC |
| **AT5G34828** | 1 | 175 | 167 | GGGTTTAGG |
| **AT5G34846** | 1 | 642 | 650 | CCTAAACCC |
| **AT5G34868** | 1 | 95 | 87 | GGGTCTAGG |
| **AT5G35380** | 1 | 655 | 663 | CCTAAACCC |
| **AT5G35390** | 1 | 767 | 759 | GGGTTTAGG |
| **AT5G35416** | 1 | 659 | 651 | GGGCTCAGG |
| **AT5G35575** | 1 | 314 | 306 | GGGTTTAGG |
| **AT5G35794** | 1 | 757 | 749 | GGGCCTAGG |
| **AT5G35890** | 1 | 734 | 726 | GGGTTTAGG |
| **AT5G36300** | 1 | 193 | 185 | GGGTTTAGG |
| **AT5G36700** | 1 | 393 | 401 | CCTAAACCC |
| **AT5G36790** | 1 | 396 | 404 | CCTAAACCC |
| **AT5G37125** | 1 | 871 | 879 | CCTAAACCC |
| **AT5G37130** | 1 | 490 | 482 | GGGTTTAGG |
| **AT5G37340** | 1 | 292 | 300 | CCTAAACCC |
| **AT5G37410** | 1 | 128 | 136 | CCTAAACCC |
| **AT5G38565** | 1 | 540 | 548 | CCTAGGCCC |
| **AT5G39590** | 1 | 393 | 401 | CCTAAACCC |
| **AT5G40395** | 1 | 384 | 392 | CCTAAACCC |
| **AT5G40440** | 1 | 738 | 730 | GGGTTTAGG |
| **AT5G40460** | 1 | 606 | 614 | CCTAAACCC |
| **AT5G40720** | 1 | 942 | 950 | CCTAAACCC |
| **AT5G40790** | 1 | 77 | 85 | CCTAAACCC |
| **AT5G42200** | 1 | 855 | 863 | CCTAAACCC |
| **AT5G42430** | 1 | 877 | 869 | GGGCCTAGG |
| **AT5G42785** | 1 | 274 | 266 | GGGTTCAGG |
| **AT5G42825** | 1 | 696 | 688 | GGGTTTAGG |

| | | | | |
|---|---|---|---|---|
| **AT5G43750** | 1 | 185 | 193 | CCTAAACCC |
| **AT5G44050** | 1 | 33 | 25 | GGGTTTAGG |
| **AT5G44100** | 1 | 552 | 560 | CCTAAACCC |
| **AT5G44894** | 1 | 333 | 325 | GGGTTTAGG |
| **AT5G44920** | 1 | 217 | 209 | GGGTTTAGG |
| **AT5G44960** | 1 | 757 | 765 | CCTAAACCC |
| **AT5G45469** | 1 | 497 | 505 | CCTAAACCC |
| **AT5G47480** | 1 | 963 | 955 | GGGTTTAGG |
| **AT5G47880** | 1 | 904 | 896 | GGGCTTAGG |
| **AT5G47890** | 1 | 923 | 931 | CCTAAGCCC |
| **AT5G48940** | 1 | 986 | 994 | CCTAAACCC |
| **AT5G49138** | 1 | 937 | 945 | CCTAAACCC |
| **AT5G49430** | 1 | 477 | 469 | GGGTTTAGG |
| **AT5G49435** | 1 | 156 | 164 | CCTAAACCC |
| **AT5G50200** | 1 | 704 | 712 | CCTAAACCC |
| **AT5G52160** | 1 | 948 | 956 | CCTAAACCC |
| **AT5G52380** | 1 | 691 | 699 | CCTAGACCC |
| **AT5G53451** | 1 | 653 | 661 | CCTAAGCCC |
| **AT5G54745** | 1 | 794 | 802 | CCTAAGCCC |
| **AT5G54750** | 1 | 960 | 952 | GGGCTTAGG |
| **AT5G55680** | 1 | 475 | 467 | GGGTTTAGG |
| **AT5G56380** | 1 | 1 | 9 | CCTAGACCC |
| **AT5G56500** | 1 | 832 | 824 | GGGTTTAGG |
| **AT5G56540** | 1 | 279 | 271 | GGGTTTAGG |
| **AT5G57970** | 1 | 701 | 693 | GGGTTTAGG |
| **AT5G57990** | 1 | 422 | 414 | GGGTTTAGG |
| **AT5G58412** | 1 | 340 | 332 | GGGTTTAGG |
| **AT5G58910** | 1 | 256 | 264 | CCTAAGCCC |
| **AT5G58920** | 1 | 298 | 290 | GGGCTTAGG |
| **AT5G60030** | 1 | 546 | 538 | GGGTTTAGG |
| **AT5G60060** | 1 | 957 | 965 | CCTAAACCC |
| **AT5G60966** | 1 | 898 | 906 | CCTGAACCC |
| **AT5G61170** | 1 | 506 | 514 | CCTGAACCC |
| **AT5G61450** | 1 | 601 | 593 | GGGCTTAGG |
| **AT5G61690** | 1 | 486 | 478 | GGGTTCAGG |
| **AT5G61750** | 1 | 23 | 15 | GGGTTCAGG |
| **AT5G61830** | 1 | 389 | 397 | CCTAAACCC |
| **AT5G63280** | 1 | 991 | 999 | CCTAAACCC |
| **AT5G63340** | 1 | 546 | 554 | CCTAGGCCC |
| **AT5G64060** | 1 | 959 | 967 | CCTAAACCC |
| **AT5G64780** | 1 | 962 | 954 | GGGCCCAGG |
| **AT5G64790** | 1 | 526 | 534 | CCTGGGCCC |
| **AT5G65290** | 1 | 430 | 438 | CCTAAACCC |

| | | | | |
|---|---|---|---|---|
| **AT5G65340** | 1 | 986 | 994 | CCTAAACCC |
| **AT5G65533** | 1 | 236 | 228 | GGGCTTAGG |
| **AT5G65540** | 1 | 951 | 959 | CCTAAGCCC |
| **AT5G66020** | 1 | 525 | 517 | GGGTTCAGG |
| **AT5G66180** | 1 | 919 | 927 | CCTAAGCCC |
| **AT5G66558** | 1 | 697 | 689 | GGGTTTAGG |
| **AT5G66562** | 1 | 709 | 717 | CCTAAACCC |
| **AT5G66564** | 1 | 558 | 566 | CCTAAACCC |
| **AT5G66567** | 1 | 419 | 427 | CCTAAACCC |
| **AT5G66760** | 1 | 157 | 149 | GGGTCCAGG |
| **AT5G66840** | 1 | 276 | 284 | CCTAAGCCC |
| **AT5G67150** | 1 | 895 | 887 | GGGCCTAGG |
| **AT5G67350** | 1 | 232 | 240 | CCTAGGCCC |
| **AT5G67430** | 1 | 285 | 293 | CCTAAACCC |
| **ATCG00610** | 1 | 101 | 109 | CCTGAACCC |
| **ATCG00620** | 1 | 362 | 370 | CCTGAACCC |
| **ATCG00640** | 1 | 715 | 707 | GGGTTCAGG |
| **ATCG00650** | 1 | 286 | 278 | GGGTTCAGG |
| **ATCG00920** | 1 | 766 | 774 | CCTAAACCC |
| **ATCG01210** | 1 | 766 | 774 | CCTAAACCC |
| **ATMG00370** | 1 | 325 | 333 | CCTAAACCC |
| **ATMG00390** | 1 | 433 | 425 | GGGTTTAGG |
| **ATMG00660** | 1 | 788 | 780 | GGGCTTAGG |
| **ATMG00665** | 1 | 996 | 988 | GGGCTTAGG |
| **ATMG00950** | 1 | 50 | 58 | CCTGGACCC |
